# The role of multiparametric resonance and biopsy in prostate cancer detection: comparison with definitive histological report after laparoscopic/robotic radical prostatectomy

**DOI:** 10.1007/s00261-020-02798-8

**Published:** 2020-10-13

**Authors:** S. Rapisarda, M. Bada, F. Crocetto, B. Barone, D. Arcaniolo, A. Polara, C. Imbimbo, G. Grosso

**Affiliations:** 1 Department of Urology, Ospedale Pederzoli, Peschiera del Garda, Verona, Italy; 2 Department of Urology, San Bassiano Hospital, Bassano del Grappa, Vicenza, Italy; 3 Department of Neurosciences, Reproductive and Odontostomatological Sciences, School of Medicine, Federico II University of Naples, Via Sergio Pansini, no 5, 80131 Naples, Italy; 4 Department of Woman, Child and General and Specialized Surgery, Urology Unit, University of Campania “Luigi Vanvitelli”, Naples, Italy

**Keywords:** Magnetic resonance imaging, Fusion biopsy, PIRADS, Concordance rate

## Abstract

**Purpose:**

Magnetic Resonance Imaging (MRI) targeted biopsy increases overall detection rates and decreases the risk of clinically insignificant PCa detection. The aim of this retrospective study is to compare concordance rates regarding side of lesion and Gleason Score at fusion targeted/systematic biopsy and MRI with the definitive histologic report of prostatectomy specimen.

**Methods:**

115 patients underwent multiparametric (mp) MRI and successively fusion targeted/systematic biopsy. 107 patients, with a positive biopsy for PCa, further underwent laparoscopic/robotic radical prostatectomy. We compared surgical histologic report with biopsy histologic report for side of lesion and Gleason Score. We further compared PIRADS score at mpMRI with Gleason Score of both histologic reports.

**Results:**

Concordance rate for mpMRI lesion side was 74% compared to biopsy and 52.3% compared to surgical histologic report (*p* < 0.0001). Fusion targeted/systematic biopsy reported a concordance rate with surgical histologic report of 67.3% for side of the lesion, while Gleason Score was concordant for 73.6% for clinically significant cancer (Gleason Score ≥ 7) (*p* < 0.0001). PIRADS score ≥ 3 was further associated with clinically significant cancer at surgical histologic report in 92.4% of cases (*p* = 0.359).

**Conclusion:**

Multiparametric MRI of the prostate reaches a good and improvable accuracy in the detection of suspicious PCa before biopsy. A combined approach of fusion targeted and systematic biopsy could further increase the overall accuracy in PCa diagnosis, especially in biopsy-naïve patients, reaching concordance rates with definitive histologic report up to 52.3% and 85.5%.

## Introduction

Prostate cancer (PCa) is the most common malignancy among European men. The incidence of PCa varies widely between different geographical areas, being highest in Oceania and Northern America, with an age-standardized rate (ASR) of 79.1 and 73.7 per 100,000 respectively, followed by Europe (ASR 62.1 per 100,000) [1]. These findings are largely related to the use of prostate specific antigen (PSA) testing and the overall increasing age [2]. PSA testing has indeed increased PCa incidence rate while decreasing the related mortality. However, PSA mass screening remains one of the most controversial topic in the urological literature due to the risks of overdiagnosis and overtreatment of clinically insignificant PCa [3–5]. Currently, diagnosis of PCa is determined by histologic report of biopsy undertaken in case of clinical suspicion, high PSA level and/or abnormal digital rectal examination (DRE). There are two standard techniques for prostate biopsy: transrectal ultrasound-guided biopsy (TRUS-GB) and trans perineal ultrasound-guided biopsy (TPUS-GB) [6, 7]. Despite the increased risks of complications (rectal bleeding, fever, sepsis, hematuria and acute urinary retention), TRUS-GB remains, however, the gold standard [8, 9]. TRUS is, indeed, ideal to guide the biopsy needle and determine prostate gland volume but lacks both sensitivity and specificity for PCa detection and staging, with further loss of sensitivity related to the heterogeneous appearance of the gland, often caused by benign prostatic hyperplasia (BPH), which makes detection of anteriorly located tumors particularly difficult [10]. On the other side, multiple biopsies may hit small clinically insignificant PCa microfocus and could contribute to overdiagnosis and increased risk of overtreatment [11]. Moreover, detection rate of TRUS re-biopsy is only 10–22% [12]. Clinical guidelines currently advise to perform an mpMRI when initial TRUS biopsy result is negative but the suspicion of PCa persists [13]. Developments of multiparametric Magnetic Resonance Imaging (mpMRI) techniques have increased the sensitivity of imaging for PCa [14], changing diagnostic and therapeutic prospects of this disease [15]. The use of 3 T (3-T) magnet compared with 1.5-T magnet [16], and the utilization of Prostate Imaging-Reporting and Data System (PIRADS) classification, introduced in 2012 by the European Society of Urogenital Radiology (ESUR) and recently updated to version 2.1 [17], have further enhanced resolution and quality of MR imaging. PIRADS score, in particular, represents a standardized method for mpMRI evaluation, developed to increase inter-reader reliability and meaningful communication towards clinicians, evaluating lesions within the prostate on each of the three imaging components (T2-weighted, diffusion weighted imaging, and dynamic contrast enhanced) using a 1–5 scale, giving additionally, for each lesion, an overall score between 1 and 5 predicting its chance of being a clinically significant cancer.

## Aim of the study

This retrospective study aims to evaluate the diagnostic accuracy of mpMRI and fusion targeted/systematic biopsy compared to definitive histologic report after radical prostatectomy. In particular, we compared side of suspicious lesion (both at mpMRI and biopsy) and Gleason score at biopsy with the definitive histologic report. Similarly, we further compared PIRADS score with biopsy and definitive histologic report.

## Materials and methods

We recruited 115 patients which underwent targeted TRUS fusion biopsy from March 2018 to September 2019 at the Hospital Pederzoli, Peschiera del Garda, Verona, Italy. We collected the following information: age, informed consent, PSA, DRE report and previous biopsies. Indications for mpMRI were based on clinical suspicion of PCa (PSA > 4 ng/ml, positive DRE). Biopsies were performed in patients with mpMRI results of PIRADS ≥ 3, persistent clinical suspicion or patients in active surveillance for a previously diagnosed low-risk PCa.

### mpMRI and biopsy protocols

mpMRI was performed using a 3-T MRI scanner Siemens *MAGNETOM Vida 3 T*, acquiring diffusion-weighted imaging (DWI), dynamic contrast enhancement imaging (DCE), T1-weighted axial and T2-weighted tri-planar imaging**.** All mpMRI images were independently interpreted by four different experienced genitourinary radiologists, with at least 5 years of experience, according to PIRADS version 2.0. The images were segmented in order to obtain and recordlesions locations and PIRADS score. Patients with lesions identified on mpMRI (PIRADS ≥ 3) underwent a combined biopsy (systematic plus fusion targeted biopsy) performed by a single urologist. Five patients, despite a negative mpMRI, underwent a systematic biopsy due to a frankly positive DRE (one patient), active surveillance protocol (two patients) and unexplained persistent increase of PSA > 4 ng/ml (two patients). All biopsies were carried out following a standardized protocol. T2-weighted axial, sagittal and coronal sequences of the mpMRI were uploaded into an MRI/US fusion device (*Hitachi Arietta v70* with integrated real-time ultrasonography) and the suspicious lesions were marked in three planes using the Real-time Virtual Sonography (RVS) software. Patients wereadministered a single dose of Sulfamethoxazole/Trimethoprim plus a periprostatic lidocaine infiltration nerve blockade before biopsy. Systematic biopsy protocol based on EAU guidelines was performed, including 12–16 cores collected in an extended-sextant template from lateral to medial of base, mid, and apex portions of the prostate on both sides. In addition, fusion targeted biopsy was performed on the previously identified mpMRI lesions with T2-weighted sequence overlapped on the real-time TRUS images. Each lesion was sampled in axial and sagittal planes. A diagrammatic report was taken per lesion for a maximum of two lesions while number of cores and histologic report was registered. We defined a clinically significant prostate cancer as any cancer with Gleason score ≥ 4 + 3 and/or any cancer occupying ≥ 6 mm of a biopsy core, in according to the PROMIS trial [18]. Patients with positive biopsy for PCa underwent robot-assisted radical prostatectomy at our institution.

### Statistical analyses

An Excel database was created to report all the previous data and the collected histologic reports of patients who underwent radical prostatectomy. All statistical analyses were conducted using SPSS software (version 25, SPSS Inc., Chicago, IL). Descriptive statistics included means and standard deviations for continuous variables while frequencies and percentages were obtained for categorical variables. Differences were analyzed with the use of one-group mean comparison t-test. Pearson’s Chi-square test was used to calculate the concordance between, respectively: laterality of target lesion at mpMRI and at biopsy; laterality of target lesion at mpMRI and at definitive histologic report; laterality at biopsy and definitive histologic report; PIRADS score and Gleason score at biopsy; PIRADS score and Gleason score at the definitive histologic report; Gleason score at biopsy and at definitive histologic report. Relation between variables were considered significant for *p* < 0.05.

## Results

A total of 106 patients who underwent mpMRI fusion/standard biopsy at our institution successively underwent robot-assisted radical prostatectomy (RARP) during the study period. Descriptive characteristics of patients’ cohorts are reported in the following table (Table 1).

**Table 1:** Descriptive characteristics of patients in the study

	Mean	Standard deviation		P value
PSA (ng/ml)	8.5643	4.71305		< 0.001
Prostate Volume (ml)	49.71	20.283		< 0.001
Number of Cores	15.14	2.243		< 0.001
	50–60 years	60–70 years	70–80 years	
Age (%)	18 (15.7)	57 (49.6)	40 (34.8)	< 0.001
	Positive	Negative	Suspicious	
DRE (%)	71 (61.7)	16 (13.9)	28 (24.3)	< 0.001
	No	Yes		
Previous biopsy (%)	100 (87)	15* (13)		< 0.001

^*^Two patients in active surveillance

### mpMRI versus biopsy report

Overall, 68.3%, 70% and 91.7% of patients with a suspicious area on, respectively, right, left and bilateral side on mpMRI, were concordant with the subsequent biopsy, for a total concordance rate of 74% (*p* < 0.0001) (Table 2). PIRADS score 3 was associated with significant clinical cancer at biopsy (GS ≥ 7) in 46.7% of cases. Similarly, PIRADS score 4 was associated at biopsy with significant clinical cancer in 82.6% of cases while PIRADS score 5 was associated at biopsy with significant clinical cancer in 83.7% of cases. Negative mpMRI was, instead, associated with significant PCa in two patients (1.7%) (Table 3). Overall, a positive mpMRI (PIRADS ≥ 3) was associated with clinically significant cancer at biopsy in 74.8% of cases at biopsy (*p* < 0.0001).

**Table 2:** Concordance between Target side at mpMRI and biopsy side

Target side (%)	Biopsy Side		
	Right	Left	Bilateral
Right	28 (68.3)	1 (2.4)	12 (29.3)
Left	5 (10)	35 (70)	10 (20)
Bilateral	1 (4.2)	1 (4.2)	22 (91.7)

**Table 3:** Concordance between PIRADS score and biopsy GS

	Biopsy report—Gleason score							
	3 + 3	3 + 4	4 + 3	4 + 4	4 + 5	5 + 4	ASAP	Negative
PIRADS (%)								
3	8 (53.3)	6 (40)	1 (6.7)	0 (0)	0 (0)	0 (0)	0 (0)	0 (0)
4	9 (17.3)	23 (44.2)	15 (28.8)	4 (7.7)	1 (1.9)	0 (0)	0 (0)	0 (0)
5	4 (9.3)	9 (20.9)	13 (30.2)	9 (20.9)	3 (7)	2 (4.7)	2 (4.7)	1 (2.3)
Negative	0 (0)	1 (20)	1 (20)	0 (0)	0 (0)	0 (0)	2 (40)	1 (1)

### mpMRI versus definitive histologic report

The laterality concordance of the lesion at mpMRI with the definitive histologic report was confirmed in 31.6%, 46.7% and 95.8% of cases for, respectively, right, left and bilateral side, for a total concordance rate of 52.3% (*p* < 0.0001) (Table 4). Concordances between PIRADS and definitive histologic report of the radical prostatectomy (RP) specimen are reported in the following table (Table 5). Regarding PIRADS 3 score, 85.7% of cases were associated with significant clinical cancer at definitive histologic report. PIRADS 4 score, similarly, was associated with significant clinical cancer at definitive histologic report in 92.4% of cases. PIRADS 5 score was, finally, associated with clinically significant cancer at definitive histologic report in 100% of cases. As previously reported, two patients of those with negative mpMRI reported a GS 7 PCa at definitive histologic report. Overall, a positive mpMRI was associated with clinically significant cancer at definitive histologic report in 92.4% of cases (*p* = 0.359).

**Table 4:** Concordance between Target side and side at final histologic report of the specimen post prostatectomy

		Specimen Side		
		Right	Left	Bilateral
Target side (%)	Right	12 (31.6)	7 (18.4)	19 (50)
	Left	4 (8.9)	21 (46.7)	20 (44.4)
	Bilateral	0 (0)	1 (4.2)	23 (95.8)

**Table 5:** Concordance between PIRADS score and GS at final histologic report of the specimen post prostatectomy

	Specimen report—Gleason score						
	3 + 3	3 + 4	4 + 3	4 + 4	4 + 5	5 + 4	5 + 5
PIRADS (%)							
3	2 (14.3)	7 (50)	3 (21.4)	2 (14.3)	0 (0)	0 (0)	0 (0)
4	4 (7.7)	20 (38.5)	16 (30.8)	11 (21.2)	0 (0)	1 (1.9)	0 (0)
5	0 (0)	7 (18.4)	14 (36.8)	11 (28.9)	2 (5.3)	3 (7.9)	1 (2.6)
Negative	0 (0)	1 (50)	1 (50)	0 (0)	0 (0)	0 (0)	0 (0)

### Biopsy report versus definitive histologic report

The laterality concordance of the lesion between biopsy and definitive histologic report was instead 50%, 57.6% and 86.4% for, respectively, right, left and bilateral side, with a total concordance rate of 67.3% (*p* < 0.0001) (Table 6). Concordances between biopsies and definitive histologic reports are reported in the following table (Table 7). Patients with GS 6 at biopsy were confirmed in 31.6% of cases, while 68.4% of cases were understaged. Patients with GS 7 at biopsy had a concordance rate of 85.5% with the definitive histologic report. Similarly, patients with GS 8 at biopsy had a concordance rate of 84.6%. Only two cases on a total of five were concordant in patients with GS 9 (40%). Overall, the concordance rate between biopsy and definitive histologic report was 73.6% (*p* < 0.0001).

**Table 6:** Concordance between biopsy side and side at final histologic report of the specimen post-prostatectomy

		Specimen side		
		Right	Left	Bilateral
Biopsy side (%)	Right	15 (50)	4 (13.3)	11 (36.7)
	Left	1 (3)	19 (57.6)	13 (39.4)
	Bilateral	0 (0)	6 (13.6)	38 (86.4)

**Table 7:** Concordance between biopsy GS and GS at final histologic report of the specimen post prostatectomy

		Specimen Report – Gleason Score						
		3 + 3	3 + 4	4 + 3	4 + 4	4 + 5	5 + 4	5 + 5
Biopsy Report – Gleason Score	3 + 3	6 (31.6)	3 (15.8)	5 (26.3)	5 (26.3)	0 (0)	0 (0)	0 (0)
	3 + 4	0 (0)	31 (79.5)	7 (17.9)	1 (2.6)	0 (0)	0 (0)	0 (0)
	4 + 3	0 (0)	1 (3.3)	20 (66.7)	6 (20)	0 (0)	3 (10)	0 (0)
	4 + 4	0 (0)	0 (0)	1 (7.7)	11 (84.6)	1 (7.7)	0 (0)	0 (0)
	4 + 5	0 (0)	0 (0)	1 (25)	1 (25)	1 (25)	0 (0)	1 (25)
	5 + 4	0 (0)	0 (0)	0 (0)	0 (0)	0 (0)	1 (100)	0 (0)

## Discussion

MpMRI is increasingly used to improve the diagnostic pathway of prostate cancer. Different studies have provided further evidence to support a larger use of mpMRI due to more accurate detection and characterization of suspicious lesions, especially in biopsy-naïve patients, reducing, moreover, the number of unnecessary biopsies [19, 20]. The PROMIS study proposed and tested mpMRI as a “triage test” to avoid unnecessary prostate biopsies and reduce diagnoses of non-clinically significant PCa, which represent, respectively, 27% and 5% of cases. Anyway, suspicious lesions detected at mpMRI still needs to be confirmed at biopsy before any further therapeutic decision, due to the possibility of incurring in false positives [21–23]. Despite fusion targeted biopsy is able to detect PCa with higher GS and/or upgrade cancers previously detected compared to standard biopsy [24], a small fraction of patients with clinically significant PCa are still missed by target biopsy, due to the failure in the identification of lesion at mpMRI, technical limitations and intralesional GS heterogenicity [25, 26]. However, the aid of mpMRI before biopsy could further enhance the detection rate also for systematic biopsy, as shown by the PRECISION trial [27] and reduce the risk of upgrading GS on radical prostatectomy [28–30]. Due to these findings, we utilized in our study a biopsy protocol that comprehended a combined approach of systematic and fusion targeted biopsy, in order to obtain the highest possible accuracy. Kam et al. [31], evaluating the accuracy of mpMRI in detecting PCa and tumor staging, reported, on 235 patients, an overall index lesion location concordance between mpMRI and RP histologic report of 75% with a sensitivity that reached 91% in the detection of clinically significant PCa (GS ≥ 7). In a previous study [32], the same authors reported, on 121 patients, a concordance rate for GS between combined biopsy (fusion plus standard) and final histologic report of 58%. However, both studies presented limitations and bias regarding blinding methods and randomization which could have increased sensitivity and concordance rates. Baco et al. [33], similarly, reported on 128 patients, a concordance rate for GS between fusion biopsy and RP histologic report of 69.5%. Borkowetz et al. [34] reported a concordance rate for GS between targeted, systematic and combined biopsy of 63%, 54% and 75%, respectively. The same study also reported a concordance rate for index lesion location between mpMRI and RP histologic report of 87% with, in addition, detection of clinically significant PCa in 54%, 88% and 96% of cases for, respectively, PIRADS 3, 4 and 5. Those higher rates could, however, be explained by the absence of blinding methods in the evaluation of mpMRI which were retrospectively re-evaluated by a single radiologist, leading, as reported also by the authors, to a detection bias in favor of an overdetection of tumor-suspicious lesions. Finally, a recent study by Diamand et al. [35], compared the accuracy in the detection of clinically significant PCa between standard and fusion biopsy, reporting concordance rates of 49.4%, 51.2% and 63.2% for, respectively, standard biopsy, fusion biopsy and both at the final histologic report of the postoperative specimen. Our data are consistent with the previously reported studies, and despite we obtained lower concordance rates for lesion locations (which could be in part explained by the absence of a dimensional cut point), our concordance rates regarding GS, between biopsy and RP histologic report, and detection of clinically significant PCa at mpMRI are even higher with 73.6% for GS concordance and 92.4% for detection of clinically significant cancer at mpMRI. Our results could, therefore, suggest, in our opinion, an extended use of mpMRI as a safe and efficient diagnostic exam for PCa, reducing the number of men to submit to biopsy. Despite those considerations, prostate biopsy remains essential for the diagnosis of PCa, with last guidelines that underline the need to perform both targeted and systematic biopsies to improve the sensitivity of biopsy itself [36]. However, there are several limitations in our study. First, our manuscript is based on retrospective analysis and even though comparable studies do not rely on larger sample sizes, our study is limited to a cohort of 115 patients. Second, despite mpMRI was executed by expert genitourinary radiologists, interpretation agreement between them was not assessed. Third, a potential bias is represented by the unavoidable discrepancies between biopsy and definitive histologic due to the multifocality of PCa. Finally, nine patients did not perform radical prostatectomy and despite this is a small fraction of total patients enrolled, this could lead to a potential selection bias.

## Conclusion

mpMRI reported concordance rates with definitive histologic reports of 52.3% and 92.4% for, respectively, side of lesion (*p* < 0.0001) and detection of clinically significant cancer (GS ≥ 7) (*p* = 0.359). Similarly, fusion targeted plus systematic biopsy reported, instead, concordance rates of 67.3% and 73.6% for, respectively, side of lesion (*p* < 0.0001) and detection of clinically significant cancer (GS ≥ 7) (*p* < 0.0001). mpMRI alone, despite its limitations, confirms its role in the identification of clinically significant PCa before biopsy. The execution of exclusively fusion targeted biopsies is not currently supported by recent guidelines due to the risk of missing clinically significant PCa, however fusion targeted biopsy, complementary to systematic biopsy, could further enhance detection rates and reduce this risk.

## Data Availability

Available on request to the corresponding author.
